# Banking of cryopreserved iliac artery and vein homografts: clinical uses in transplantation

**DOI:** 10.1007/s10561-014-9469-2

**Published:** 2014-08-24

**Authors:** Wee Ling Heng, Krishnakumar Madhavan, Priscilla Wee, Tracy Seck, Yeong Phang Lim, Chong Hee Lim

**Affiliations:** 1 National Cardiovascular Homograft Bank, National Heart Centre Singapore, Singapore, Singapore; 2 National University Centre for Organ Transplantation, National University Hospital, Singapore, Singapore

**Keywords:** Liver transplantation, Allografts, Artery, Vein, Cryopreservation

## Abstract

Iliac artery and vein homografts are critical for revascularization in living-donor liver transplantation. Since 2010, National Cardiovascular Homograft Bank and National University Hospital have collaborated in the pioneer endeavor of banking iliac vessel homografts for such surgeries in Singapore. This article aims to demonstrate that the processing, decontamination and cryopreservation techniques that our bank follow, help preserve iliac vessel homografts for a longer duration as compared to homografts preserved using short-term preservation techniques. This paper reports the first 4 years of post-operative outcome for recipients as a preliminary report for a longer-term outcome study. Criteria for donor assessment, techniques of iliac vessel homograft recovery, processing, decontamination, cryopreservation and storage according to the American Association of Tissue Banks standards are also described. From 2010 until 2013, we discovered of the iliac vessel homografts processed, 17 (94.4 %) were suitable for clinical use. Nine iliac artery grafts (64 %) and one iliac vein graft (14 %) were implanted. Irrespective of vessel type, homografts <90 mm in length were of little use. Of the nine current iliac vessel homograft recipients, eight patients (89 %) had living-donor liver transplantation and one patient (11 %) had reconstruction of the right internal carotid artery after resection of an aneurysm. Our preliminary results supports existing literatures that suggest cryopreserved iliac vessel homografts can be successfully used for revascularization in liver transplantation and reconstruction of carotid artery. Encouraging short-term post-operative patient outcomes have been achieved, with no report of adverse event attributed to implanted homografts. We believe that our processing, decontamination and cryopreservation techniques have helped preserve the homografts for longer duration as compared to homografts preserved using short-term preservation techniques.

## Introduction

Artery or vein homografts are frequently used for vascular reconstruction in liver transplantation (Martínez et al. 1999). Recovery of vascular grafts was first recommended by Starzl et al. as a life-saving measure for an unexpected emergency; it provides additional vessel length for the recipient during organ transplantation (Starzl et al. 1979). This recovery is important because life-threatening complications that affect the hepatic artery after transplantation can result in ischemia of the liver graft, causing graft failure or even patient mortality (Vivarelli et al. 2004a, b). In most cases, vascular grafts, such as iliac vessels, are recovered together with the liver from the same deceased donor and used for the same recipient. However, iliac artery or vein homografts can also be used when the liver recipient of a living donor develops late hepatic artery aneurysm/pseudo-aneurysm or when autologous vein grafts or deceased organ donor’s artery/vein homografts are unavailable (Sellers et al. 2002).

Before banking of cryopreserved iliac vessel homografts started, liver transplant surgeons relied on other techniques of vascularization. These included the use of fresh or short-term preserved iliac vessels from liver donors (Starzl et al. 1979; Martínez et al. 1999; Sellers et al. 2002; Muralidharan et al. 2004), autologous vein grafts (Hwang et al. 2005), allogeneic cryopreserved vein grafts such as saphenous veins (Kuang et al. 1996), and even cryopreserved descending aortic conduit for one of our liver recipients, who was diagnosed with liver cirrhosis caused by Budd-chiari syndrome.

Different methods for the short-term preservation of vascular homografts have been reported. Short-term preservation is useful for emergency cases which require the repair of post-transplant vascular complications in the recipient. Martinez et al. stored iliac vessel homografts in Terasaki solution (McCoys lymphocyte culture medium, bovine fetal serum, HEPES buffer, gentamicin) at 4 °C for 1–26 days (Martínez et al. 1999). Sellers et al. treated the vessels with RPMI-1640 with l-glutamine containing antibiotics (240 ug/mL cefoxitine, 120 ug/mL lincomycin, 50 ug/mL vancomycin and 100 ug/mL polymixin B) at 4–10 °C for 24 h. They were subsequently kept in fresh RPMI-1640 with 2.05 l-glutamine without antibiotics, and stored at 4–10 °C for a maximum of 30 days (Sellers et al. 2002). Other studies mentioned the preservation of fresh iliac arteries using University of Wisconsin solution at 4 °C for <10 days (Shames et al. 2003; Ma et al. 2011). Alternative preservation media, such as Celsior solution, Euro-Collins solution and histidine-tryptophan-ketoglutarate (HTK) solution have also been reported (Vivarelli et al. 2004a, b). In addition, a few literatures described the long-term preservation processes: for instance, Mabrut et al. decontaminated their iliac vessels with antibiotic solution containing lincocine (120 ug/mL), mefoxin (200 ug/mL), colimycin (1,000 IU/mL) and vancomycin (50 ug/mL) (Mabrut et al. 2012). The European Homograft Bank used a different antibiotic regimen consisting of vancomycin, lincomycin and polymixin B, with incubation temperature of 4 °C for a duration of 24 h (Jashari et al. 2013). For both centres, 10–15 % dimethyl sulfoxide (DMSO) was used as a cryoprotectant, and cryopreservation was achieved through controlled rate freezing (Mabrut et al. 2012; Jashari et al. 2013). However, despite the clinical relevance, a paucity of literature exists regarding the use of cryopreserved iliac vessels and their clinical outcome (Kuang et al. 1996; Vivarelli et al. 2004a, b; Mabrut et al. 2012).

Recognizing the critical importance of iliac vessel homografts for vascularization of liver recipients in living-donor transplantation, the National Cardiovascular Homograft Bank (NCHB) and the National University Centre for Organ Transplantation have collaborated in the pioneer endeavor of banking iliac artery and vein homografts for such surgeries in Singapore since 2010. Apart from liver transplantation, iliac vessel homograft has also been used by our hospital for surgical emergencies in life-threatening conditions.

This article is a description of our routine processes in recovery, processing, decontamination and cryopreservation techniques of iliac artery and vein homografts in compliance to the American Association of Tissue Banks (AATB) standards. A retrospective review of post-operative outcome for homograft recipients as a preliminary study of a longer-term outcome study is also presented.

## Materials and methods

### Donor assessment

Donors are multi-organ donors aged 12–66 years. Evaluation of donor suitability is conducted by the clinical coordinator. This includes performing a physical assessment of the potential donor, reviewing medical records and the medical social history questionnaire. The potential donor is excluded if he/she had engaged in high-risk social behavior and/or is suspected or tested positive for transmissible diseases (AIDS, hepatitis B or C, syphilis, dengue fever, active tuberculosis), sepsis, malignancies or autoimmune diseases.

He/she is also deemed unsuitable if his/her blood is suspected to be hemodiluted, as this will interfere with the accuracy of the infectious diseases test results. Plasma hemodilution can occur either when the potential donor experiences extensive blood loss or when he/she receives a transfusion of significant volumes of whole blood, erythrocytes, colloids and/or crystalloids prior to cross-clamp. The donor’s blood is defined as hemodiluted when (1) the total volume of crystalloids transfused in the previous 1 h and colloids transfused in the previous 48 h is greater than the donor’s total plasma volume; or (2) the total volume of crystalloids transfused in the previous 1 h and the total volume of colloids and blood products transfused in the previous 48 h is greater than the donor’s total blood volume.

### Homograft recovery, preservation and storage

Stringent aseptic techniques are upheld during the tissue recovery and processing procedures to prevent microbial and fungal contamination of the tissues. First, iliac artery and/or vein homografts are recovered by the liver surgeon during multi-organ recovery in the operating theatre. After retrieval, bench work is performed immediately. This involves the measurement of vessel length and diameter, and rinsing the tissues in cold saline (0.1–10 °C) to remove any blood clots. Vessels are also inspected for patency and the presence of sclerosis or trauma. Vessels which are too short (<90 mm) or display signs of degenerative, sclerotic or traumatic abnormalities are discarded. As the AATB has mandated a standardized evaluation and classification system for homografts, the vessels’ conditions are evaluated and classified into three categories: (1) tissue with no visible abnormalities, (2) tissue with imperfections and (3) non-implantable tissue. Tissues in the first two categories are deemed morphologically acceptable upon initial assessment and are transported in cold HTK solution to the NCHB laboratory for further processing.

As stipulated by the AATB (2012) requirements, the total ischaemic time must be less than 48 h (AATB, 13th edition). Processing of tissues is performed in an ISO Class 5 laminar flow hood located in an ISO Class 7 cleanroom. The laminar flow hood work surface is sterile and draped according to normal surgical procedure. Before bio-burden reduction, the iliac vessel homografts are rinsed in three rounds of saline. They are then decontaminated using the same antibiotic regimen that the NCHB adopted for disinfecting cardiovascular homografts, which uses amikacin (100 ug/mL) and vancomycin (50 ug/mL) in Medium 199 (M199). The incubation condition is 2–8 °C for 24–28 h. After antibiotic disinfection, the vessels are rinsed in fresh M199 without antibiotics for 12 min to remove any residual antibiotics that may remain on the tissues.

Finally, the tissue is packaged individually in a sterile cryogenic bag that contains the freeze solution (10 % DMSO in M199). Prior to heat-sealing, air is removed from the bag to prevent rupture of the bag during temperature changes while thawing. The sealed bag is then inserted into a slightly larger cryogenic bag and heat-sealed, creating a double-layer package for additional sterility protection of the homografts. Controlled rate freezing of the processed homograft is attained by operating a tissue freezing profile using a controlled rate freezer. The rate of cooling is approximately −1 °C per minute. The transfer of the homograft to a quarantine liquid nitrogen storage tank takes place when the temperature probe of the freezing surrogate package reaches −50 °C.

### Quality control

For the safety of recipients, the results of infectious disease tests and microbiological cultures of tissue and solution specimens are evaluated. A donor is determined to be unsuitable if the results of infectious disease tests are positive, with the exception of the venereal disease research laboratory (VDRL) test. When the VDRL result is reactive, a confirmatory treponema pallidum hemagglutination or treponema pallidum particle agglutination test is performed to exclude active syphilis infection. Although some banks exclude donors with both previous and active syphilis due to the potential histological damage caused to the arteries by the infection, AATB stated that tissue from a donor reactive of syphilis using U.S. Food and Drug Administration (FDA)-approved non-treponemal screening assay may be used for transplantation only if the donor’s blood sample is tested negative using FDA-approved treponemal-specific confirmatory assay (AATB, 13th edition). Hence, due to the severe shortage of homografts, NCHB only rejects donors with active syphilis to maximise the number of implantable tissues.

The testing of total antibodies to hepatitis B core antigen test (anti-HBc total) is also mandatory. If the anti-HBc total is positive, an additional test for hepatitis B surface antibodies (anti-HBs) is performed to rule out current hepatitis B infection. The acceptable anti-HBs level is ≥10 μ/mL. In addition, a dengue polymerase chain reaction test and a *Mycobacterium tuberculosis* culture are also performed for donors who have suspected infection, due to the prevalence of these diseases in Southeast Asia.

Microbiological cultures for aerobes, anaerobes and fungi are performed on tissue and solution specimens after recovery, post-antibiotic incubation, and before freezing. Cryopreserved iliac vessel homografts are stored under quarantine until all of the infectious disease and microbiological results confirm their clinical suitability. The NCHB’s quality assurance staff checks and verifies all results and donor records before submitting them for final review to the Medical Director. After review, clinically suitable homografts are transferred to a clinical liquid nitrogen storage tank and made available for transplantation. Cryopreserved tissues are stored in liquid nitrogen vapor for duration of 5 years.

### Thawing and implantation

The homograft is shortlisted by the transplant surgeon based on its length. As homografts are mostly used in the reconstruction of segments 5 and 8 veins from right liver graft where patency is required only for about 3 weeks, ABO blood group compatibility is not mandated.

On the day of transplant, the selected graft is transported to the operating theatre of the transplant hospital in a cryoshipper at a temperature below −135 °C. After the transplant surgeon approves the thawing of homograft, the cryopreserved package containing the tissue is immersed in saline pre-warmed to approximately 37–42 °C. Saline of higher temperatures is not used; although rapid thawing of the homograft package can be achieved, this sudden temperature difference will increase the risk of breakage of the homograft package, thereby rendering the tissue non-sterile and non-implantable. After complete thawing, a circulating nurse cuts the outer freezing package. The sterile inner package containing the homograft is then handled by the scrub nurse. Subsequently, the homograft is rinsed in three rounds of lactated Ringers or HTK solution for 5 min each to remove the antibiotics and DMSO residues. Finally, it is kept in lactated Ringers or HTK solution in a sterile closed bottle. If the immediate transplantation is not achieved, the bottle containing the homograft will be placed either on a tray of ice or in the 4 °C refrigerator.

The final step of quality assurance involves the testing of post-thaw tissue and solution specimens for microbiological and fungal cultures. If the post-thaw result is positive, it will be treated as an adverse event regardless of the cause of contamination. The transplant surgeon will be notified immediately so that a prompt follow-up on the recipient and appropriate medical intervention can be administered.

### Ethical consideration

The iliac vessel donors are either tissue pledgers or donation is consented from the next-of-kin for deceased donors under the Ministry of Health Singapore’s (MOH) Medical (Therapy, Education and Research) Act. The MOH’s guidelines on Code of Ethical Practice in Human Biomedical Research and Helsinki declaration guidelines were consulted. This study did not expose the donors or recipients to additional risk or discomfort because this article is a description of our routine processes and a retrospective review of post-operative outcome. Furthermore, since there was no identification of patients in this article and patient confidentiality was strictly observed, hence ethics approval was advised to be unnecessary by our hospital.

## Results

### Processing activity outcome

Iliac artery and vein grafts have been recovered from six anatomical locations: (1) left iliac arteries, (2) right iliac arteries, (3) common and bilateral iliac arteries, (4) left iliac veins, (5) right iliac veins, and (6) bilateral iliac veins. Their lengths ranged from 90 to150 mm; vessels which do not fulfil the minimum length of 90 mm are of little use to the recipients. To date, all the 17 iliac vessel homografts suitable for clinical use have been evaluated and classified to be “tissue with no visible abnormalities”.

The program ceased temporarily in year 2012. As a result, no homograft recovery or implantation was facilitated during this period. In 2010, there was an equal number of iliac artery and vein grafts recovered. However, owing to the low demand for iliac vein homografts, the number of vein grafts recovered in year 2013 declined to 17 % of the total iliac grafts recovered. In contrast, the number of iliac artery homografts recovered increased. For two donors, a pair of iliac arteries was procured after the surgeons confirmed that the donors’ vessels were not required for the liver recipients. In total, nine iliac artery grafts (64 %) and one iliac vein graft (14 %) were implanted (Table 1).

**Table 1:** The number of iliac vessel homografts recovered versus the number of homografts implanted each year

Iliac vessel homograft	Year 2009		Year 2010		Year 2011		Year 2013	
	No. of grafts recovered	No. of grafts implanted	No. of grafts recovered	No. of grafts implanted	No. of grafts recovered	No. of grafts implanted	No. of grafts recovered	No. of grafts implanted
Artery	2	1 (50 %)	4	4 (100 %)	1	1 (100 %)	7	3 (43 %)
Vein	1	0 (0 %)	4	1 (25 %)	0	0 (0 %)	2	0 (0 %)

A review of infectious disease and microbiological results revealed that 17 (94.4 %) iliac vessel homografts processed was suitable for clinical use. The initial contamination rate of post-recovery homografts was 27.7 % (5 homografts). 80 % (4 homografts) of these grafts were successfully decontaminated by the current antibiotic regimen. A fungus *Malassezia furfur* was isolated post-recovery in one iliac vein homograft. Although it was subsequently tested negative for post-incubation culture, it was still discarded due to the pathogenicity of the micro-organism. Other common bacteria that had been isolated post-recovery included coagulase-negative Staphylococcus and non-methicillin-resistant *Staphylococcus aureus* (Table 2). The former is a common skin commensal, while the latter is frequently found in the human respiratory tract.

**Table 2:** Types of micro-organisms isolated from iliac artery and vein homografts

	Total no. of homografts	Post-recovery tissue or solution culture		Post-incubation tissue or solution culture	
		No. of homografts with positive culture	Type of micro-organisms isolated/No. of homografts	No. of homografts with positive culture	Type of micro-organisms isolated
Iliac artery homograft	12	4	Coagulase-negative Staphylococcus (2) Non-methicillin- resistant *Staphylococcus aureus* (2)	0	–
Iliac vein homograft	6	1	*Malassezia furfur*	1	–

### Recipients and outcome

Eight (89 %) iliac vessel homograft recipients were patients who underwent living-donor liver transplantation. Of these, one recipient received a pair of iliac artery grafts from the same donor. An elderly patient who was diagnosed with a right mycotic internal carotid artery aneurysm also received an iliac vessel homograft. As a case of surgical emergency, he urgently required a vessel for reconstruction of the right internal carotid artery after resection of the aneurysm (Table 3). All the recipients recovered and are doing well post-operatively.

**Table 3:** Clinical diagnosis of the iliac vessel homograft recipients and the type of surgery

Recipient No.	Type of homograft used	Diagnosis	Surgery
1	Iliac artery graft	Primary sclerosing cholangitis	Living-donor liver transplantation
2	Iliac artery graft	Liver cirrhosis secondary to autoimmune hepatitis	
3	Iliac artery graft	Hepatitis C liver cirrhosis	
4	Iliac artery graft	Acute liver failure secondary to hepatitis B flare	
5	Iliac artery graft	Hepatitis C liver cirrhosis	
6	Iliac artery graft	Cryptogenic liver cirrhosis (possibly NASH-induced)	
7	Iliac artery graft	Hepatitis B flare	
8	Iliac vein graft	Cryptogenic liver cirrhosis	
9	Iliac artery graft	Right mycotic internal carotid artery aneurysm	Reconstruction of right internal carotid artery after resection of aneurysm

## Discussion

This collaborative program has seen a higher demand for iliac artery homografts as compared to iliac vein homografts. This is because there are more advantages to using an iliac artery homograft, which includes (1) it has a thicker wall, and can retain its length, axis and shape after restoration of blood flow in the hepatic vessels, (2) the probable benefit of decreasing the incidence of early anastomotic stenosis, (3) the surgical technique for iliac artery graft is easier than for iliac vein graft, and (4) its short-term patency rate was comparable to that of an iliac vein graft (Hwang et al. 2005). We also learned that irrespective of vessel type, homografts of a length <90 mm were of little use. As a result, the homografts of shorter lengths recovered in 2009 during the initial phase of our bank’s operation had to be removed from the inventory of transplantable tissues after they had reached the 5-years of maximum cryopreservation shelf-life. Henceforth, to maximize the probability of graft utilization, iliac vessel homografts of as great a length as possible (≥100 mm) will be procured.

Not all liver transplant patients require the implantation of an iliac vessel homograft during surgery. Previously, the absence/thrombosis of the portal vein in the recipient or abnormal hepatic arteries in the donor were contraindications to liver transplantation. However, these problems were overcome by the use of iliac artery or vein grafts from the donor. This technique has since become standard procedure (Strong 2001). It has also been discovered that recipients who have been on long-term steroid therapy pre-transplantation are more susceptible to developing a diseased wall in the hepatic artery. This is a result of steroid-induced angiopathy related to hypercholersteremia and the diabetogenic effect of steroids (Khalaf et al. 2007). For such recipients, iliac vessel homografts should always be made available during surgery. The main advantage of using cryopreserved homografts is their availability for elective surgeries. As such, it is especially beneficial for living-donor liver transplant patients. Unlike cadaveric donor liver transplant patients who can receive the liver and fresh iliac vessel from the same deceased donors, living-donor liver transplant patients have no option of receiving fresh homografts for revascularization when artery complications are discovered peri-operatively. They can only rely on cryopreserved homografts. Usually, liver transplant surgeons can only ascertain whether iliac vessel homograft is required after assessing the condition of the recipient’s vessels during surgery. Therefore, as a cost-saving measure for the recipients, our bank is activated for homograft transportation and thawing only on the day of surgery. This is usually done after the surgeons determine if there is a requirement for the reserved homograft.

The significance of ABO blood group incompatibility as a risk factor that results in early homograft degeneration has been controversial. Mostly, correlations are postulated from the analysis of post-operative graft and recipient outcomes. Experiences in organ allotransplantation have shown that donor-recipient blood group incompatibility elicits a strong rejection response. Therefore, blood group cross-matching is performed in many centers (Kadner et al. 2001). In contrast, there are centers in which donor-recipient blood-group matching is considered to be unnecessary (Hwang et al. 2005; Mabrut et al. 2012). Justification derived from immunological studies, such as the one conducted by Kadner et al. who reported that cryopreserved homografts did not appear to possess an endothelial layer, as observed from a lack of CD31 expression. Moreover, the blood group antigens were not detected on cryopreserved homografts that had been thawed, unlike fresh tissues in which the strong expression of the antigens was detected (Kadner et al. 2001). In addition, Martinez et al. reported that the actuarial liver transplant survival rates for recipients of ABO-incompatible vessels were not statistically different from recipients of matched fresh vessels (Martínez et al. 1999). Finally, for organ recipients who are already on immunosuppressive therapy, the therapy will abrogate their immune response to the donor grafts (Shaddy and Hawkins 2002). This explains why allografts may function as well as autografts (Sellers et al. 2002) and suggests that donor-recipient blood-group matching might not be required. The implication of removing the requirement for ABO compatibility between recipient and donor homografts is an increase in the availability of cryopreserved homografts for the recipients (Mabrut et al. 2012).

Despite encouraging short-term results, which revealed a 100 % survival rate and no cases of graft failure, infection or adverse events attributed to the implanted homograft among our recipients, the long-term post-operative outcome should be further studied. The long-term outcome appears to vary depending on the type of complication (thrombosis or stenosis), timing of the arterial disease (early or late presentation with respect to the time of transplantation) (Vivarelli et al. 2004a, b), the indications for liver transplantation (Khalaf et al. 2007) and the promptness of diagnosis (Vivarelli et al. 2004a, b). The initial technical success rates and long-term graft survival appear to be promising (Martínez et al. 1999; Del Gaudio et al. 2005; Khalaf et al. 2007; Jashari et al. 2013). For example, Khalaf et al. reported that the survival rate of the grafts and patients at 5 years post-transplantation was approximately 80–90 %, with a 10-year survival rate of approximately 75 % (Khalaf et al. 2007). Del Gaudio et al. reported that 1-, 3- and 5-year overall survival were approximately 70 % each (Del Gaudio et al. 2005). Liver recipients for autoimmune hepatitis were reported to show exceptionally encouraging long-term outcomes, with patient and graft survival at approximately 90 % (Khalaf et al. 2007). In contrast, Kuang et al. reported that in his institution, retrospective analysis of long-term cryopreserved iliac vein graft performance (≥3 years) in portal vein reconstruction in living-donor liver transplantation revealed a high rate of late graft failures (Kuang et al. 1996). In addition, Del Gaudio et al. suggested that retransplantation, donor age and iliac artery conduit were significant risk factors contributing to poor graft survival and high incidence of early hepatic artery thrombosis (Del Gaudio et al. 2005).

In our context, long-term follow-up is challenging. Firstly, our number of both donors and recipients are very small, which makes an accurate and representative study of long-term outcome difficult. Secondly, some of our foreign recipients are no longer on follow-up with our hospital after discharge. Thirdly, the causes of iliac vessel graft failure and patient survival are multifactorial and difficult to predict due to the complications of liver transplantation (in particular, liver rejection and the extent and effects of immunosuppression) which might affect the iliac vessels’ graft performance and outcome (Khalaf et al. 2007).

In conclusion, our preliminary results supports existing literatures that suggest iliac vessel homografts, especially the artery grafts, can be successfully used for arterial revascularization in living-donor liver transplantation and emergency surgery involving reconstruction of the right internal carotid artery. Encouraging short-term post-operative patient and graft outcomes have been achieved, with no clinically significant infection or adverse event attributed to the cryopreserved implanted homograft observed. Therefore, based on this study of the first 4 years of post-operative outcome for recipients, although long-term patency of the homografts remains unknown, we believe that our processing, decontamination and cryopreservation techniques help to preserve iliac vessel homografts for a longer duration as compared to homografts preserved using short-term preservation techniques as previously described.

## References

[CR1] American Association of Tissue Banks (2012) Standards for Tissue Banking 13th edition

[CR2] Del Gaudio M, Grazi GL, Ercolani G, Ravaioli M, Varotti G (2005). Outcome of hepatic artery reconstruction in liver transplantation with an iliac arterial interposition graft. Clin Transplant.

[CR3] Hwang S, Lee SG, Ahn CS, Park KM, Kim KH (2005). Cryopreserved iliac artery is indispensable interposition graft material for middle hepatic vein reconstruction of right liver grafts. Liver Transplant.

[CR4] Jashari R, Van Hoeck B, Ngakam R, Goffin Y, Fan Y (2013). Banking of cryopreserved arterial allografts in Europe: 20 years of operation in the European Homograft Bank (EHB) in Brussels. Cell Tissue Bank.

[CR5] Kadner A, Chen RH, Mitchell RN, Adams DH (2001). Homograft crossmatching is unnecessary due to the absence of blood group antigens. Ann Thorac Surg.

[CR6] Khalaf H, Mourad W, El-Sheikh Y, Abdo A, Helmy A (2007). Liver transplantation for autoimmune hepatitis: a single-center experience. Transplant Proc.

[CR7] Kuang AA, Renz JF, Ferrell LD, Ring EJ, Rosenthal P (1996). Failure patterns of cryopreserved vein grafts in liver transplantation. Transplantation.

[CR8] Ma Y, Li Q, Ye ZM, Zhu XF, He XS (2011). Use of arterial conduit for arterial revascularisation during liver and multivisceral transplantation. Chin Med J.

[CR9] Mabrut JY, Abdullah SS, Rode A, Bourgeot JP, Eljaafari A (2012). Cryopreseved iliac artery allograft for primary arterial revascularization in adult liver transplantation. Clin Transplant.

[CR10] Martínez JA, Rigamanti W, Rahier J, Gigi J, Lerut J (1999). Preserved vascular homograft for revascularisation of paediatric liver transplant. Transplantation.

[CR11] Muralidharan V, Imber C, Leelaudomlipi S, Gunson BK, Buckels JAC (2004). Arterial conduits for hepatic artery revascularisation in adult liver transplantation. Transpl Int.

[CR12] Sellers MT, Haustein SV, McGuire BM, Jones C, Bynon JS (2002). Use of preserved vascular homografts in liver transplantation: hepatic artery aneurysms and other complications. Am J Transplant.

[CR13] Shaddy RE, Hawkins JA (2002). Immunology and failure of valved allografts in children. Ann Thorac Surg.

[CR14] Shames BD, Odorico JS, D’Alessandro AM, Pirsch JD, Sollinger HW (2003). Surgical repair of transplant renal artery stenosis with preserved cadaveric iliac artery grafts. Ann Surg.

[CR15] Starzl TE, Halgrimson CG, Koep LJ (1979). Vascular homografts for cadaveric organ donors. Surg Gynecol Obstet.

[CR16] Strong RW (2001). Liver transplantation: current status and future prospects. J R Coll Surg Edinb.

[CR17] Vivarelli M, Cavallari A, Buzzi M, Conte R (2004). Successful arterial revascularization in liver transplantation using a cryopreserved arterial allograft. Transplantation.

[CR18] Vivarelli M, Cucchetti A, La Barba G, Bellusci R, De Vivo A (2004). Ischemic arterial complications after liver transplantation in the adult: multivariate analysis of risk factors. Arch Surg.

